# Effects of Aging and Nerve Growth Factor on Neuropeptide Expression and Cholinergic Innervation of the Rat Basolateral Amygdala

**DOI:** 10.3390/biology13030155

**Published:** 2024-02-28

**Authors:** Pedro A. Pereira, Marta Tavares, Miguel Laires, Bárbara Mota, Maria Dulce Madeira, Manuel M. Paula-Barbosa, Armando Cardoso

**Affiliations:** 1 Unit of Anatomy, Department of Biomedicine, Faculty of Medicine, University of Porto, Alameda Professor Hernâni Monteiro, 4200-319 Porto, Portugal; up201807431@edu.med.up.pt (M.T.); mlairestrc@gmail.com (M.L.); caetanomota@gmail.com (B.M.); madeira@med.up.pt (M.D.M.); insanato@med.up.pt (M.M.P.-B.); cardosoa@med.up.pt (A.C.); 2 NeuroGen Research Group, Center for Health Technology and Services Research (CINTESIS), Rua Dr. Plácido da Costa, 4200-450 Porto, Portugal; 3 CINTESIS@RISE, Faculty of Medicine, University of Porto, Alameda Professor Hernâni Monteiro, 4200-319 Porto, Portugal

**Keywords:** aging, basolateral amygdala, cholinergic innervation, nerve growth factor, neuropeptide Y, vasoactive intestinal polypeptide

## Abstract

**Simple Summary:**

The basolateral amygdala (BLA) contains neurons that produce neuropeptide Y (NPY) and vasoactive intestinal polypeptide (VIP). These neuropeptides are involved in the regulation of several brain functions and behaviors, and there is evidence that, in some brain regions, the acetylcholine might modulate its expression. Thus, we study if aging alters the BLA densities of NPY- and VIP-positive neurons and cholinergic varicosities. We also investigate if any potential change in these parameters might be related to insufficient trophic support provided by the neurotrophin nerve growth factor (NGF). We found that the density of NPY-positive neurons was reduced in aged rats, whereas the density of VIP-immunoreactive neurons was unchanged. The decreased expression of NPY was fully reversed by intracerebroventricular administration of NGF. The density of cholinergic varicosities was similar in adult and old rats, and the infusion of NGF to aged rats increased their density to greater than the values of adult and old rats. These results indicate that the NPY expression in the BLA is affected by aging, and also show that this age-related change can be associated with alterations in the NGF trophic support. Furthermore, the results also show that the BLA cholinergic innervation is particularly resistant to age effects.

**Abstract:**

The basolateral amygdala (BLA) contains interneurons that express neuropeptide Y (NPY) and vasoactive intestinal polypeptide (VIP), both of which are involved in the regulation of functions and behaviors that undergo deterioration with aging. There is considerable evidence that, in some brain areas, the expression of NPY and VIP might be modulated by acetylcholine. Importantly, the BLA is one of the brain regions that has one of the densest cholinergic innervations, which arise mainly from the basal forebrain cholinergic neurons. These cholinergic neurons depend on nerve growth factor (NGF) for their survival, connectivity, and function. Thus, in this study, we sought to determine if aging alters the densities of NPY- and VIP-positive neurons and cholinergic varicosities in the BLA and, in the affirmative, if those changes might rely on insufficient trophic support provided by NGF. The number of NPY-positive neurons was significantly reduced in aged rats, whereas the number of VIP-immunoreactive neurons was unaltered. The decreased NPY expression was fully reversed by the infusion of NGF in the lateral ventricle. The density of cholinergic varicosities was similar in adult and old rats. On the other hand, the density of cholinergic varicosities is significantly higher in old rats treated with NGF than in adult and old rats. Our results indicate a dissimilar resistance of different populations of BLA interneurons to aging. Furthermore, the present data also show that the BLA cholinergic innervation is particularly resistant to aging effects. Finally, our results also show that the reduced NPY expression in the BLA of aged rats can be related to changes in the NGF neurotrophic support.

## 1. Introduction

The brain is particularly vulnerable to aging and undergoes significant morphological, neurochemical, and functional changes during this process [1,2,3,4,5,6,7]. The cholinergic system is among the brain neurochemical systems most vulnerable to aging, and there are data that support an association between changes in this system and the functioning of the brain [8,9,10,11,12,13,14]. There is evidence that acetylcholine is involved in the regulation of essential functions of the organism, such as motor control, motivation, cognition, sleep–wake cycles, regulation of neuropeptide expression in the brain, reward, and addiction [13,15,16,17,18,19,20,21,22,23,24,25]. One of the main recognized subdivisions of the brain cholinergic system is the basal forebrain cholinergic system [23,26], and it is well known that the basal forebrain cholinergic neurons (BFCN) depend on nerve growth factor (NGF) for their survival, connectivity, and function [27,28]. Importantly, there is also evidence that aging is associated with disruption of the neurotrophic support provided by NGF [28,29,30] and reduction in the number of BFCN [9,10,11] and that NGF delivered to the brain can revert the age-associated alterations in the number of BFCN [10,11].

One of the brain regions that has one of the densest cholinergic innervations is the basolateral amygdala (BLA), which arises mainly from the BFCN [26,31,32,33,34,35,36]. In this context, it is also important to highlight that there is evidence of the existence of neurons immunoreactive for choline acetyltransferase (ChAT) in several nuclei of the rat amygdala [32]. The BLA is one of the groups of nuclei of the amygdala [37,38,39]. The amygdala is a brain structure that is a central component of neural circuits that regulate, for instance, learning and memory, affective behavior, fear, anxiety, stress, reward behavior, and addiction [40,41,42,43,44]. There are data showing that aging is associated with changes in the BLA, both in rodents [45,46] and humans [47]. The BLA is a cortical-like structure, and thus, similarly to other cortical regions, its neuronal population is formed mostly by excitatory glutamatergic neurons (80–85%), and by a heterogeneous group of gamma-aminobutyric acid (GABA)ergic interneurons [38,39,48,49]. Both groups of neurons are postsynaptic targets of BFCN terminals [50]. With respect to the BLA interneurons, it is known that they can be divided into several groups based on their intrinsic properties, such as morphological, immunohistochemical, and electrophysiological ones [38,39,44,48,49]. Previous immunohistochemical studies in rats have demonstrated that different subpopulations of these interneurons express, amongst other neuropeptides, the neuropeptide Y (NPY) and the vasoactive intestinal polypeptide (VIP) (for review see [38,48]). These two neuropeptides are involved in several brain functions and behaviors, such as cognition, endocrine regulation, feeding, and the modulation of circadian rhythms (for a review see [51,52,53,54,55]). It is important to highlight that there is evidence that, in some brain regions, the expression of these neuropeptides might be modulated by acetylcholine [19,21,22,24,56,57]. Additionally, it is also known that the expression of these peptides is reduced in aging [12,57,58,59,60] and that the intracerebroventricular administration of NGF can revert the age-related decrease in their expression [12,57,59,60].

Despite the great knowledge about the effects of aging on the brain, what is known about the effects of aging on the different GABAergic populations of the amygdala as well as on the amygdala cholinergic innervation is still scarce. Thus, the present study was designed to examine whether there are age-associated changes in the densities of NPY- and VIP-positive neurons and cholinergic varicosities in the BLA of Wistar male rats and, in the affirmative, if those alterations can be reversed by intracerebroventricular administration of NGF. With this purpose, we have also estimated the above-mentioned parameters in the BLA of old rats that were treated, during the last 12 days of the experiment, with NGF delivered into the right lateral ventricle.

## 2. Materials and Methods

### 2.1. Animals and Treatments

Adult (6 months, n = 5) and old (24 months, n = 10) male Wistar rats were used in the present study. The animals were housed in a temperature-controlled room (22 °C) in 12 h light/dark cycles (lights on at 7:00 a.m.) with free access to a solid diet (4RF21/C; Mucedola, Milan, Italy) and tap water. Half (n = 5) of the aged rats were randomly selected and infused intracerebroventricularly with 2.5S NGF (Prince Laboratories, Toronto, ON, Canada) during the last 12 days of life (details are described in Section 2.2). Since there is evidence from our and from other laboratories that intracerebroventricular injection of vehicle does not interfere with the central NPY [57], VIP [56,61,62], and cholinergic [63,64,65,66] systems, vehicle-treated rats were not included in the present study. Additionally, because it was previously demonstrated that the intracerebroventricular infusion of NGF to young rats does not alter the brain expression of NPY [57] and VIP [59], a group of NGF-treated young rats was not included in this study.

All the experiments of this study were performed in accordance with the European Communities Council Directive of 22 September 2010 (2010/63/EU) and Portuguese Act nº129/92. All efforts were made in order to minimize the number of used rats, as well as their suffering and discomfort.

### 2.2. Surgical Procedures and NGF Treatment

The intracerebroventricular infusion of NGF was performed as previously described in detail [12,56,57,60,62,63,67]. Briefly, the rats were deeply anesthetized by sequentially injecting, at intervals of 10 min, solutions of promethazine (10 mg/kg body weight, subcutaneous; Laboratórios Vitória, Amadora, Portugal), followed by xylazine (2.6 mg/kg body weight, intramuscular; Sigma-Aldrich Company Ltd., Madrid, Spain) and, lastly, ketamine (50 mg/kg body weight, intramuscular; Merial Portuguesa, Rio de Mouro, Portugal). Then, the rats were placed on a stereotaxic apparatus with bregma and lambda in the same horizontal plane. After this step, a midline skin incision was made and the calvaria were exposed. For the delivery of NGF, permanent stainless steel cannulae (Alzet brain infusion kit; Alza Corporation, Palo Alto, CA, USA) were stereotaxically positioned in the right lateral ventricle, 1.7 mm lateral to the midline, 1.1 mm posterior to the bregma, and 4.0 mm below the skull surface [68]. Then, the cannulae were connected to methylene blue (0.01%; Sigma)-filled Alzet osmotic minipumps (model 2002; Alza Corporation, Palo Alto, CA, USA) via sterile coiled polyethylene tubing (PE-60; Intramedic, Becton Dickinson, Sparks, MD, USA). The mentioned tubing was filled with an air–oil spacer at the pump end and with NGF (150 μg diluted in 150 μL of vehicle). The vehicle was composed of artificial cerebrospinal fluid supplemented with 0.1% bovine serum albumin (Sigma). The osmotic minipumps, which were pretested to confirm their delivery rate, were implanted subcutaneously in the neck of the rats. The skin incisions were closed with surgical stitches and then were treated with local antiseptic. After surgical procedures, the animals were individually housed and maintained in a warm place until they woke up. To prevent dehydration and weight loss, postoperative care consisted of subcutaneous injections of 0.9% saline (2 mL) during the 48 h after surgery. Twelve days after the beginning of NGF administration, rats were euthanized and the total volume infused was calculated. The mean volume of NGF injected per animal was 117.27 ± 29.35 μL, and the mean flow rate of the osmotic minipumps was 0.41 ± 0.10 μL/h. The correct positioning of each cannula was confirmed in the proper sections of the brain of each animal after its processing (immunohistochemistry and Giemsa staining).

### 2.3. Tissue Preparation

At the end of the experiment, the rats were anesthetized by intraperitoneal injection of a solution (3 mL/kg body weight) containing 1% sodium pentobarbital and 4% chloral hydrate in physiological saline. Then, the rats were perfused transcardially with 150 mL of 0.1 M phosphate buffer (PB; pH 7.6) for vascular rinse, followed by 250 mL of a fixative solution containing 4% paraformaldehyde in PB (pH 7.6). The brains were removed from the skulls, coded, and postfixed for 1 h in the same fixative. Then, they were maintained overnight in a solution of 10% sucrose in PB, at 4 °C. The blocks containing the anterior part of the BLA were placed on a vibratome and serially sectioned in the coronal plane at 40 μm throughout their length. The obtained sections were collected in phosphate-buffered saline (PBS), and, after that, transferred into de Olmos cryoprotectant solution where they were stored at −20 °C until further processing. From the entire set of sections collected from each block, 4 series were formed by using a systematic, random sampling method [69,70]. Accordingly, the first section was randomly selected from the first group of 4 collected sections, and the remaining were sampled at regular intervals of 160 μm (i.e., 1 out of 4 sections). The first, second, and third series of sections were used for NPY, VIP, and vesicular acetylcholine transporter (VAChT) immunostaining, respectively, whereas the fourth was used for staining with Giemsa.

### 2.4. Immunohistochemistry and Giemsa Staining

Immunohistochemical procedures were performed based on the methodology previously described [59,60,62,71]. Specifically, sections were washed twice in PBS and treated with 3% H_2_O_2_ for 10 min to inactivate endogenous peroxidase. In order to increase tissue penetration, 0.5% Triton X-100 was added to the PBS used in all washes and immunoreactions. For visualization of NPY- and VIP-positive neurons, sections were then incubated overnight at 4 °C with the primary antibody against NPY (T-4070, Bachem Ltd., Merseyside, UK; 1:10,000) or VIP (kindly supplied by Dr. Arja Sluiter, Netherlands Institute for Neuroscience, Amsterdam, The Netherlands; 1:2500). Biotinylated goat anti-rabbit antibody (Vector Laboratories, Burlingame, CA, USA; 1:400) was used as the secondary antibody. For VAChT immunostaining, firstly the sections were immersed in a 5% solution of rabbit normal serum (Vector) in PBS for 30 min at room temperature. Then, the sections were incubated with the primary antibody (AB1578, Chemicon, Millipore Corporation, Bedford, MA, USA; 1:15,000) for 72 h at 4 °C. As the secondary antibody, a biotinylated rabbit anti-goat antibody (Vector; 1:400) was used. After incubation with the secondary antibodies, sections were treated with avidin–biotin peroxidase complex (Vectastain Elite ABC kit, Vector; 1:800). In the last two steps, the incubation was carried out at room temperature for 1 h. Then, sections were incubated for 10 min in 0.05% diaminobenzidine (DAB; Sigma) to which H_2_O_2_ was added to a final concentration of 0.01%. Sections were rinsed with PBS for at least 30 min between each different step. Sections were then mounted on gelatin-coated slides, air-dried, dehydrated in a series of ethanol solutions (50%, 70%, 90%, and 100%), cleared in xylol, and coverslipped using Histomount (National Diagnostics, Atlanta, GA, USA). To prevent variability in staining, the sections from the different groups were processed in parallel. The same procedure was followed for control sections, which were incubated with PBS instead of the primary antibodies; no immunostaining was found in these sections.

**Figure 1:**
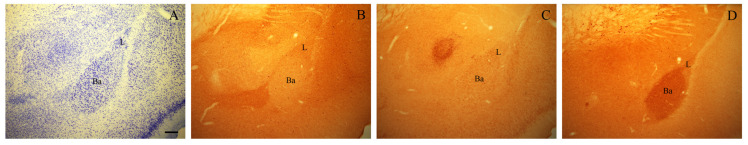
Photomicrographs of adjacent coronal sections through the rostral part of the BLA of an adult rat stained with Giemsa (**A**) and immunostained for NPY (**B**), VIP (**C**), and VAChT (**D**). Scale bar: 200 μm in (**A**–**D**). Ba, basal nucleus; L, lateral nucleus.

Sections selected for Giemsa staining (Figure 1A) were mounted on gelatin-coated slides, air-dried, stained with Giemsa (Merck, Darmstadt, Germany), dehydrated, and coverslipped with Histomount (National Diagnostics). These sections were used whenever necessary to help identify the boundaries of the BLA in immunostained sections.

### 2.5. Estimation of the Areal Densities

The estimates of the areal densities of NPY- and VIP-positive interneurons and cholinergic varicosities for each rat were obtained from an average of 12 sections. Level-matched sections of the most rostral part of the BLA including the lateral and the basal nuclei were used for all studied groups.

#### 2.5.1. NPY- and VIP-Immunoreactive Neurons

**Figure 2:**
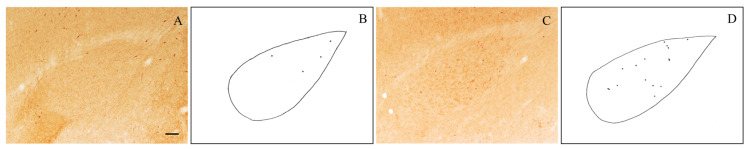
Representative photomicrographs of level-matched NPY-immunostained (**A**) and VIP-immunostained (**C**) brain sections through the rostral part of the BLA of an old rat. Camera lucida drawings of these sections are shown, respectively, in (**B**,**D**). Scale bar: 100 μm in (**A**,**C**).

The sections (Figure 1B,C) were analyzed using a light microscope equipped with a *camera lucida* at a final magnification of 133×. NPY- and VIP-positive neurons were identified as darkly stained perikarya. In each immunostained section, the profiles of NPY- and VIP-immunoreactive neuronal perikaryal as well as the boundaries of the BLA were drawn using a *camera lucida*. The number of NPY- and VIP-positive neurons within the BLA was counted from the drawings (Figure 2). The BLA area was estimated by using a transparent sheet bearing a test system composed of a set of regularly spaced points, as previously described [57,67]. The obtained cell counts were divided by the values of the corresponding areas to yield the values of the areal densities (number/mm^2^).

#### 2.5.2. VAChT-Immunoreactive Varicosities

As previously described in detail [12,57,58,67], the VAChT-immunoreactive varicosities were estimated using a computer-assisted image analyzer (Leica QWin-V3.3.1) fitted with a Leica DMR microscope and a Leica DC 300F video camera (Leica Microsystems Wetzlar GmbH, Germany), at final magnification of 1000×. In each section (Figure 1D), six different placements of the frame were randomly selected in order to obtain a mean count for the BLA. The cholinergic varicosities were identified as darkly stained axonal dilations with a size greater than 0.25 μm^2^ [12,57,58,67,72]. A sample frame (3.86 × 10^3^ μm^2^) was laid over each field of view and the number of cholinergic varicosities falling within the frame was counted. The data are expressed as areal densities (number/mm^2^).

### 2.6. Statistical Analyses

Statistical analyses were performed using the GraphPad Prism 9 (GraphPad Software Inc., San Diego, CA, USA). The data were analyzed by one-way analysis of variance (ANOVA), using treatment as the independent variable, followed by, whenever appropriate, pair-wise post hoc comparisons with the Tukey HSD test. Differences were considered to be significant if *p* < 0.05.

## 3. Results

### 3.1. Density of NPY-Immunoreactive Neurons

**Figure 3:**
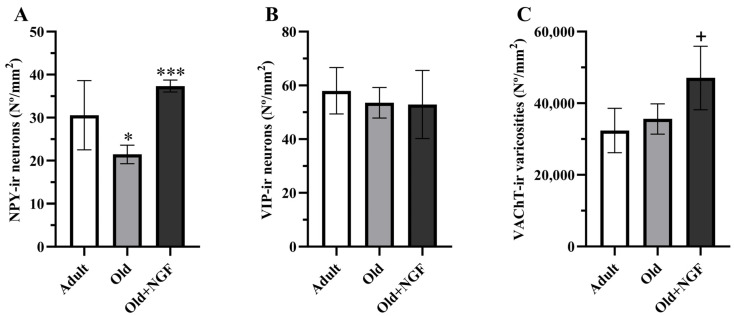
Graphic representation of the morphometric data obtained from the BLA of adult (Adult), old (Old), and NGF-treated old (Old+NGF) rats. Columns represent means and vertical bars ± 1 SD. (**A**) Density of NPY-immunoreactive neurons. The number of NPY-immunoreactive neurons is significantly reduced in old relative to adult rats. In NGF-treated old rats, the number of NPY-immunoreactive neurons recovered to levels that did not differ significantly from that of adult rats. (**B**) Density of VIP-immunoreactive neurons. The density of VIP-immunoreactive neurons does not differ between groups. (**C**) Density of VAChT-immunoreactive varicosities. No significant differences were found in the density of cholinergic varicosities between adult and old rats. The density of VAChT-immunoreactive varicosities is significantly higher in the BLA of NGF-infused aged rats than in the BLA of adult and old rats. Tukey’s post hoc tests: * *p* < 0.05, compared with adult rats; *** *p* < 0.001 compared with old rats; ^+^ *p* < 0.05, compared with adult and old rats.

**Figure 4:**
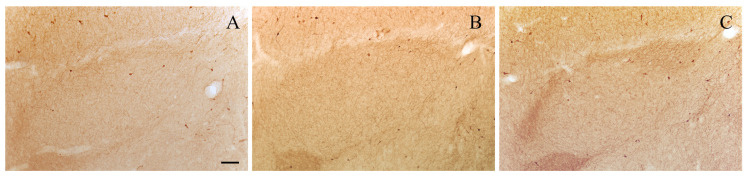
Representative photomicrographs of level-matched coronal sections of the BLA from adult (**A**), aged (**B**), and NGF-treated aged (**C**) rats immunostained for NPY. The density of NPY-immunoreactive cells in the BLA is reduced in old relative to adult rats. In NGF-treated aged rats, the density of NPY-immunostained neurons is similar to that of adult rats. Scale bar: 100 μm in (**A**–**C**).

ANOVA revealed an effect of treatment in the density of NPY-immunoreactive neurons (F(2,12) = 13.38; *p* < 0.001). Post hoc analysis showed that in aged rats the density of NPY-immunoreactive neurons was significantly reduced (*p* < 0.05) by approximately 30%. Treatment of old rats with the neurotrophin NGF was associated with a significant increase (*p* < 0.001) in the density of NPY-positive neurons to values similar to those found in adult rats (Figure 3A and Figure 4).

### 3.2. Density of VIP-Immunoreactive Neurons

Age and NGF treatment had no effect on the density of VIP-containing neurons in the BLA (F(2,12) = 0.430; *p* = 0.660; Figure 3B). According to our data, the BLA of adult rats contains approximately twice as many neurons that express VIP than neurons expressing NPY (Figure 3A,B).

### 3.3. Density of VAChT-Immunoreactive Varicosities

**Figure 5:**
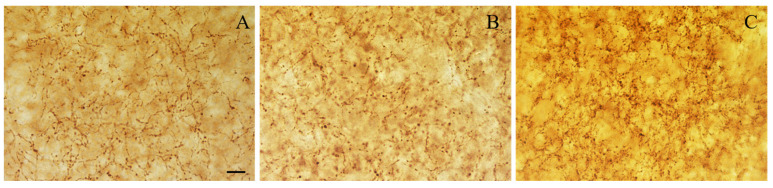
Representative photomicrographs of level-matched coronal sections of the BLA from adult (**A**), aged (**B**), and NGF-treated aged (**C**) rats immunostained for VAChT. The density of VAChT-immunoreactive varicosities is similar in adult and aged rats. The density of VAChT-positive varicosities is higher in the NGF-infused aged rats than in adult and aged rats. Scale bars: 10 μm in (**A**–**C**).

Regarding the BLA cholinergic innervation, the density of VAChT-positive varicosities was influenced by age and NGF treatment (F(2,12) = 6. 611; *p* < 0.05, Figure 3C). Actually, the density of cholinergic varicosities was similar in adult and old rats, and the infusion of NGF to aged rats significantly increased (*p* < 0.05) the density of the VAChT-immunoreactive varicosities in the BLA by 45% and 32% in relation to the values estimated in adult and old rats, respectively (Figure 3C and Figure 5).

## 4. Discussion

Herein, we examined age-associated effects on NPY and VIP expression by the BLA interneurons and their possible interrelationship to cholinergic innervation and NGF neurotrophic support. Our results show that in the BLA of aged Wistar rats there is a marked reduction in the density of NPY-immunoreactive interneurons, but not in the density of VIP-positive interneurons, and also show that the administration of NGF fully reverses the reduction in the density of NPY-producing neurons in aged rats.

Our data allow us to infer that the age-associated reduction in the density of NPY-positive interneurons detected in the present study is not due to cell death because it was found that the intracerebroventricular administration of NGF to aged rats restores their number to normal adult values. This observation is in line with previous data showing that the number of neurons in the basolateral nucleus of the amygdala does not differ between rats at 3 months of age and at 19–22 months of age [73]. Thus, it is very probable that the age-associated reduction in the NPY expression in the BLA may just reflect a decrease in the synthesis and, subsequently, in the NPY expression to levels below immunocytochemistry detectability. This hypothesis seems to be in line with results from other studies showing lower levels of NPY messenger RNA in the hypothalamus of aged rats when compared with adult rats [74,75,76,77]. Indeed, the effects of aging and exogenous NGF in the levels of NPY found in the present study were previously observed in other rat brain regions, namely in the somatosensorial cortex [57], nucleus accumbens [12], and medial prefrontal cortex [60].

On the other hand, the density of VIP-positive neurons does not undergo significant age-related alterations in the BLA. These results indicate that these neurons react to aging differently from the NPY neurons, suggesting a greater resistance of the VIP neuronal population to the effects of aging. Remarkably, this greater resistance of the VIP-immunoreactive neurons when compared to the NPY-immunoreactive neurons was also previously observed in the medial prefrontal cortex of cognitively impaired aged Wistar rats [60]. It is important to note that the NPY- and VIP-expressing cells in the BLA belong to different classes of interneurons (for review see [38]), and thus, might indeed display different vulnerabilities to normal aging. Actually, in the BLA, the neurons that express NPY are a subpopulation of interneurons that also express calbindin and somatostatin, whereas the majority of neurons that are VIP-positive are a subpopulation of interneurons that express calretinin, and, some of them, also cholecystokinin (for review see [38]). In line with our results, a reduction in the number of the calbindin interneurons in the hippocampus of aged male Fischer 344 rats was previously demonstrated that was more pronounced than in other calcium-binding protein-positive interneurons [78].

Relative to the cholinergic system, our study revealed that the density of VAChT-positive varicosities does not differ significantly between adult and old rats. Nevertheless, the results found in the literature regarding the effects of aging on the amygdala cholinergic innervation are discrepant. In fact, whereas some authors have found a reduction in the density and staining of acetylcholinesterase- and ChAT-positive fibers and terminals in the amygdala of aged Wistar rats [79], others have reported that there was no decline in the density of ChAT-positive varicosities and fibers when middle-aged human subjects were compared with senescent subjects [80]. This discrepancy might be ascribed to differences in the methodology and/or species studied. However, it is worthwhile to emphasize that in studies using VAChT as a marker of cholinergic varicosities, no changes were found in the density of these varicosities in the medial prefrontal cortex of cognitively impaired aged Fischer [81] and Wistar [60] rats, as well as in the nucleus accumbens [12] and the nucleus of the lateral olfactory tract [82] of aged Wistar rats. Conversely, in the primary somatosensory barrel field cortex [57] and in the ventral tegmental area [83] of old Wistar rats the density of VAChT-positive varicosities was found to be reduced relative to that observed in young rats. The presence of a region specificity concerning the effects of age on the cholinergic system of the brain [8,9,80,81,84] is strongly supported by our data showing a particular resistance of the BLA cholinergic innervation to aging effects.

Moreover, still regarding the cholinergic varicosities, we have found that the infusion of NGF to old rats significantly increased their density by 45% and 32% in relation to the values estimated in adult and old rats, respectively. The effect of NGF herein observed in the cholinergic varicosities is not unique because it was already demonstrated in several brain areas. Actually, it was reported that exogenous NGF increased the density of cholinergic varicosities to greater than control values in the cerebral cortex of rats submitted to unilateral devascularizing cortical lesions [85], in the nucleus accumbens of aged rats [12] and in the dentate hilus of rats submitted to prolonged alcohol withdrawal [67].

It is known that in several brain regions, the neuronal expression of NPY might be dependent on acetylcholine [19,21,22,24,56,57]. For instance, lesions of BFCN lead to a decrease in the number of NPY-positive neurons in the neocortex [24] and hippocampal formation [21] of the rat, and in the number of hypothalamic suprachiasmatic nucleus neurons expressing VIP [19]. Recently, we have also demonstrated that in the rat medial prefrontal cortex the activity of NPY-positive interneurons, but not the activity of interneurons expressing VIP, is downregulated in rats submitted to an excitotoxic lesion of the laterodorsal tegmental nucleus [22]. However, in the present study, we found no correlation between the density of NPY-immunoreactive neurons and the density of VAChT-immunoreactive varicosities in non-infused aged rats. Notwithstanding, this observation is not sufficient to exclude the involvement of the central cholinergic system in the observed effects of aging on BLA interneurons that contain NPY as they might rely on age-related alterations in other cholinergic key players. As a matter of fact, it is well known that the high-affinity choline uptake transporter is critical for the normal synthesis of acetylcholine, and thus for proper cholinergic neurotransmission (for review see [86,87]), and there are studies showing age-associated reductions in the brain high-affinity choline uptake or in the [3H]hemicholinium binding to high-affinity choline uptake sites [66,88,89,90,91,92]. In the same vein, the decrease in the density of muscarinic receptors that occurs in the amygdala during aging [93] may also underlie or, at least, contribute to the alterations we observed in the NPY expression, since there is evidence that the in vitro activation of these receptors stimulates NPY biosynthesis [94,95].

Interestingly, in the present study, we have found that the intracerebroventricular administration of NGF to old rats increased the density of NPY-positive neurons to adult values. Bearing in mind that previous studies have revealed that in the amygdala there are no cell bodies immunoreactive for NGF trkA [96] or p75 [97] receptors, the NGF-associated increase in the density of NPY-containing neurons in the BLA of old rats can only be explained by an NGF indirect action. Several hypotheses can be put forward to explain this effect on BLA levels of NPY. For example, it might result from the NGF-induced facilitation of cholinergic neurotransmission between the nucleus basalis and the amygdala in rats [98]. Actually, we found that NGF administration to aged rats significantly increased the density of cholinergic varicosities in the BLA. Furthermore, there is evidence that non-cholinergic neurons of the basal forebrain project to the basolateral nuclear complex of the rodent amygdala [31,99]. Thus, it is also possible that BFCN activated by NGF stimulates the non-cholinergic neurons of this region [100], thereby indirectly increasing the activity of the amygdala NPY-containing neurons. There is also the possibility that amygdala NPY-positive neurons might be activated by increased local dopamine release, consequent to stimulation of mesencephalic dopaminergic neurons by NGF [101].

## 5. Conclusions

Briefly, the present data show that the NPY expression by BLA interneurons is downregulated in old rats and that this age-related effect is entirely reversed by intracerebroventricular infusion of NGF, suggesting that the reduced NPY expression in the BLA of aged rats can be related to changes in NGF trophic support. Another relevant finding of this study is that the age effect on the expression of NPY is not directly related to alterations in the density of cholinergic varicosities. Indeed, our data also show that the BLA cholinergic innervation is particularly resistant to the effects of age. The results of our study might be of importance for better understanding the role of BLA interneurons, acetylcholine, and NGF in the functions attributed to the amygdala, as well as the possible role of the neurotrophin NGF in the treatment of age-associated changes in such functions.

## Data Availability

Data are available upon reasonable request to the corresponding author.

## Abbreviations

The following abbreviations are used in this manuscript:

BFCN	basal forebrain cholinergic neurons
BLA	basolateral amygdala
ChAT	choline acetyltransferase
GABA	gamma-aminobutyric acid
NGF	nerve growth factor
NPY	neuropeptide Y
VAChT	vesicular acetylcholine transporter
VIP	vasoactive intestinal polypeptide
